# Trends in the Incidence of Nasopharyngeal Cancer in Saudi Arabia Across One Decade (2007 to 2016)

**DOI:** 10.7759/cureus.24732

**Published:** 2022-05-04

**Authors:** Abdualrahman F Kabli, Khalil F Miyajan, Abdulmohsen S Alqurashi, Ammar K Mandili, Revan M Mujahed, Bayan F Hafiz, Roaa M Mandora, Ameen Z Herabi

**Affiliations:** 1 Medicine and Surgery, Department of Medicine, Faculty of Medicine, Umm Al-Qura University, Makkah, SAU; 2 Medicine, Department of Medicine, Faculty of Medicine, Umm Al-Qura University, Makkah, SAU; 3 Otolaryngology - Head and Neck Surgery, Faculty of Medicine, Umm Al-Qura University, Makkah, SAU; 4 Otolaryngology - Head and Neck Surgery, International Medical Center, Jeddah, SAU; 5 Otolaryngology - Head and Neck Surgery, King Faisal Specialist Hospital & Research Center, Jeddah, SAU

**Keywords:** head and neck surgery, saudi arabia, incident rate, cancer, nasopharyngeal cancer (npc)

## Abstract

Background

Cancer is an ongoing global health concern; it is the greatest cause of mortality in the industrialized world and the second-highest cause of death in the developing world. This study aims to assess the incidence and geographic distribution of nasopharyngeal cancer between 2007 and 2016 in Saudi Arabia.

Methods

Data between 2007 and 2016 from Saudi Cancer Registry reports were collected in this study. These reports provide information on all cancer cases, including the age, sex, geographic location, and year of diagnosis for each patient.

Result

Between 2007 and 2016, the Saudi Cancer Registry identified 110,075 cancer cases in total. The mean age-standardized rate of all cancer types for women was 51.7 compared with 48.2 for men. The percentage of cases of nasopharyngeal cancers was 1.2% for women and 2.2% for men in 2007. This percentage decreased to 0.8% for women and increased to 2.7% for men in 2016 in comparison to all cancer cases. The curve for nasopharyngeal cancer of all cancer types for men and women correlated with rises and drops in men over the study period, and a minor decrease in women over time, until another rise in 2016. A positive correlation was observed between nasopharyngeal cancer incidence and age. The age-standardized rate data for nasopharyngeal cancer cases demonstrated a wide variation across Saudi regions. The age-standardized rate per 100,000 people from 2007 to 2016 ranged from 0.39 in Jazan to 1.92 in Qassim, with a national average of 1.06.

Conclusion

From 2007 to 2016, the overall trend of the age-standardized rate for men fluctuated while the female rate slightly dropped before rising again. On the contrary, the incidence of nasopharyngeal cancer varies by region in Saudi Arabia. Further study of this variation would help focus awareness campaigns on the most susceptible regions.

## Introduction

Cancer is an ongoing global health concern; it is the greatest cause of mortality in the industrialized world and the second-highest cause of death in the developing world [1]. Nasopharyngeal cancer is an epithelial tumor that develops in the pharyngeal recess of the nasopharynx and is one of the most frequent head and neck cancers, with high prevalence rates in Asia [2]. More than 80% of patients with nasopharyngeal cancer are from Southern China and Southeast Asia [3]. In Saudi Arabia, head and neck cancers represent 5% of all malignancies diagnosed every year [4]. Nasopharyngeal cancer accounts for 31.4% of head and neck cancers diagnosed in Saudi Arabia each year, accounting for 2.7% of all malignancies in men and 0.8% in women, making it the number one head and neck cancer diagnosed in Saudi Arabia in 2016 [4-6].

Overall, nasopharyngeal cancer can occur in all age groups, but there is a bimodal age distribution with a high incidence between the ages of 50 and 60. Small peaks are observed in late childhood [7]. The etiology of nasopharyngeal cancer is distinct and multifactorial, involving genetic, epigenetic, and environmental factors such as Epstein-Barr virus infection. There is also a strong association between alcohol consumption and exposure to tobacco smoke [3,8-10]. It is challenging to diagnose nasopharyngeal cancer at an early stage since it frequently runs quietly or with vague symptoms [11]. In the late stages, its complications include swollen lymph nodes around the neck, blood in the nose and saliva, hearing loss, and ear infection. In addition, severe nasopharyngeal cancer can lead to a variety of associated neurological symptoms, including unilateral deafness and difficulty opening the mouth (known as Trotter’s syndrome) [12]. In this study, we performed an observational descriptive epidemiological analysis of nasopharyngeal cancer to help future attempts to reduce its incidence and death. We used Saudi Cancer Registry (SCR) data to assess the incidence and mortality of nasopharyngeal cancer in Saudi Arabia between 2007 and 2016 [13].

## Materials and methods

Data

A descriptive epidemiological analysis of nasopharyngeal cancer cases detected in Saudi Arabia from January 2007 to December 2016 was carried out using data from the SCR, a population-based registry established by Saudi Arabia’s Ministry of Health in 1992. The SCR’s primary objective is to collect, register, and disseminate high-quality data on cancer incidence stratified by region, age group, and year of diagnosis. The statistical reports on cancer incidence in Saudi Arabia are publicly accessible and can be obtained from the SCR website. SCR reports are available to governments, cancer researchers, treating clinicians, and cancer control and prevention organizations. No data were provided from 1994 to 2000, and the SCR’s most recent published report was in 2016.

To evaluate cancer patterns in the Saudi population, the researchers used SCR data from the International Classification of Diseases, 11th revision (ICD-11) from both men and women. The SCR has been providing reports on cancer patterns in Saudi Arabia since 2001 to determine the disease’s population-based incidence. Each of Saudi Arabia’s 13 administrative regions has its own complete report. Cancer incidence and mortality rates (i.e., the crude incidence rate and age-specific incidence rate (AIR) from 2007 to 2016 are shown in each report. Our current study gathered all information on nasopharyngeal cancer from SCR reports to describe the epidemiology of nasopharyngeal cancer in Saudi Arabia.

Data analysis

The Statistical Package for the Social Sciences (SPSS 25; BM Corporation, Armonk, NY) was used for data analysis and graph formatting. Descriptive analyses were conducted by calculating the mean number of cases, nasopharyngeal cancer percentages, AIR of age groups, and age-standardized rate (ASR) by region for 2007-2016. In the SCR reports, the crude incidence rate is defined as the number of cases relative to the total population (100,000 individuals). In contrast, the AIR is the number of cases by age and sex during a specific period divided by a midyear population of the same age and sex groups. The ASR summarizes the rate of cases if a population had the standard age structure [4]. These standardizations are essential for comparing populations that differ in number and age group.

## Results

Increase in the number of nasopharyngeal cancer cases

**Figure 1:**
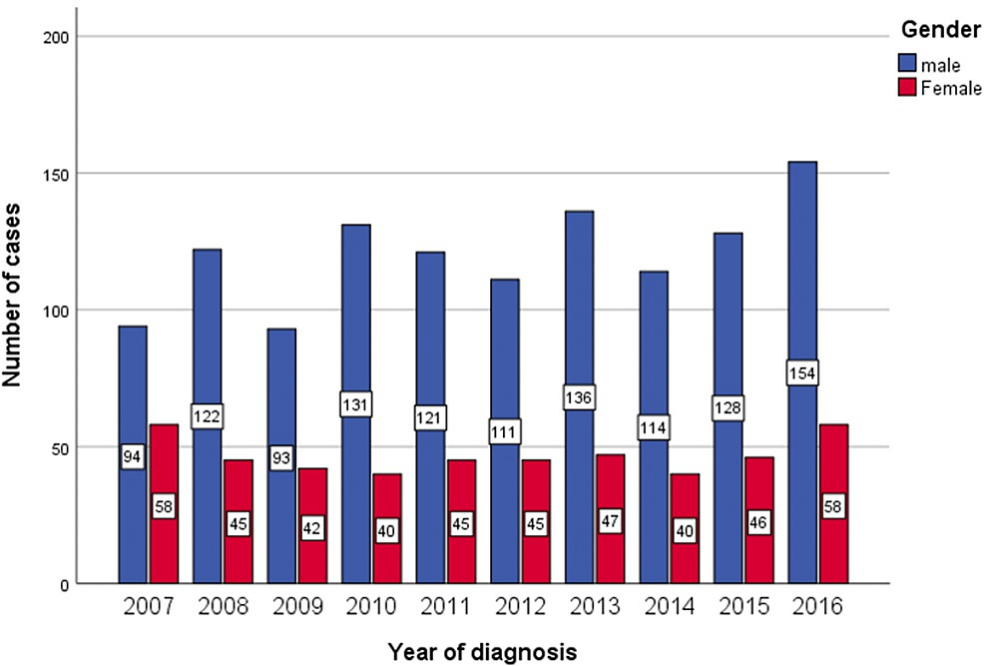
Number of nasopharyngeal cancer cases in Saudi Arabia between 2007 and 2016

From 2007 to 2016, the overall number of cancer cases recognized by the SCR in the Saudi population was 110,839, with 50,971 (45.98%) in men and 59,868 (54.01%) in women. Of these, 1670 cases (1.5%) were nasopharyngeal cancer. The number of reported cases of nasopharyngeal cancer gradually increased from 152 (94/58 M/F) in 2007 to 212 (154/58 M/F) in 2016 (Table 1 and Figure 1).

**Table 1:** Demonstrates the number of cases and the percentage of nasopharyngeal cancer compared to other cancer types in Saudi Arabia between 2007 and 2016

Year of diagnosis	Gender	Number of cases	Percentage
2007	male	94	2.2
	female	58	1.2
2008	male	122	2.9
	female	45	1
2009	male	93	2
	female	42	0.8
2010	male	131	2.9
	female	40	0.7
2011	male	121	2.5
	female	45	0.8
2012	male	111	2.2
	female	45	0.8
2013	male	136	2.6
	female	47	0.7
2014	male	114	2.2
	female	40	0.7
2015	male	128	2.3
	female	46	0.7
2016	male	154	2.7
	female	58	0.8
Overall mean	male	120.4	2.45
	Female	46.6	0.82

**Figure 2:**
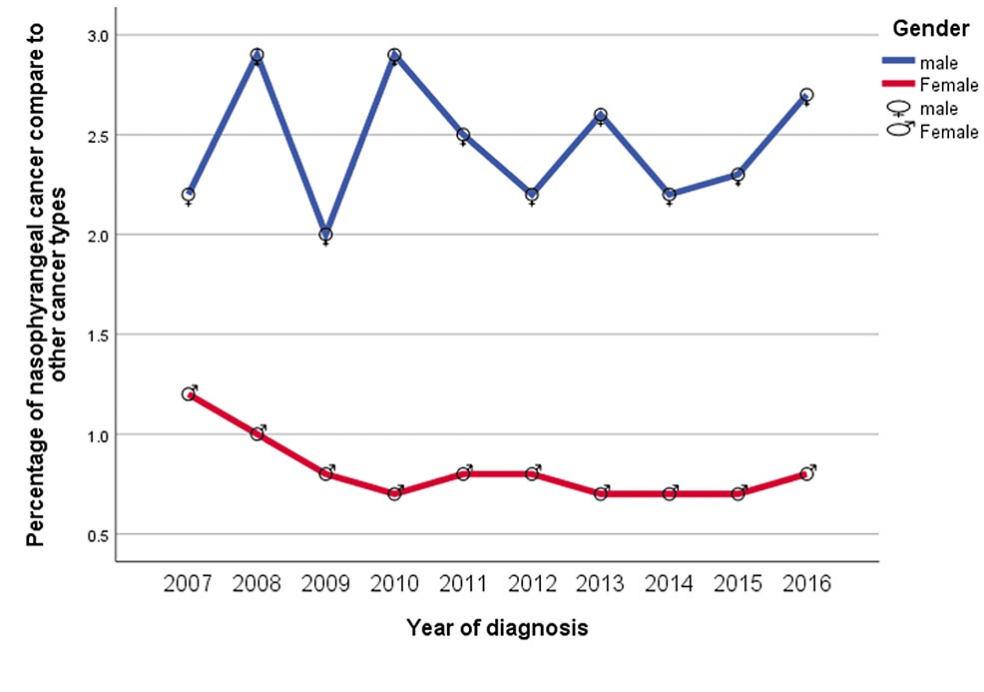
Percentage of nasopharyngeal cancer compared to other cancer types

The percentage of cases of nasopharyngeal cancers was 1.2% for women and 2.2% for men in 2007 (Figure 2). This percentage decreased to 0.8% for women and increased to 2.7% for men in 2016 (Figure 2). The curve for nasopharyngeal cancer of all cancer types for men and women correlated with rises and drops in men over the study period, and a minor decrease in women over time, until another rise in 2016 (Figure 2).

Age-standardized rate (ASR) of nasopharyngeal cancer fluctuated over the study period

**Figure 3:**
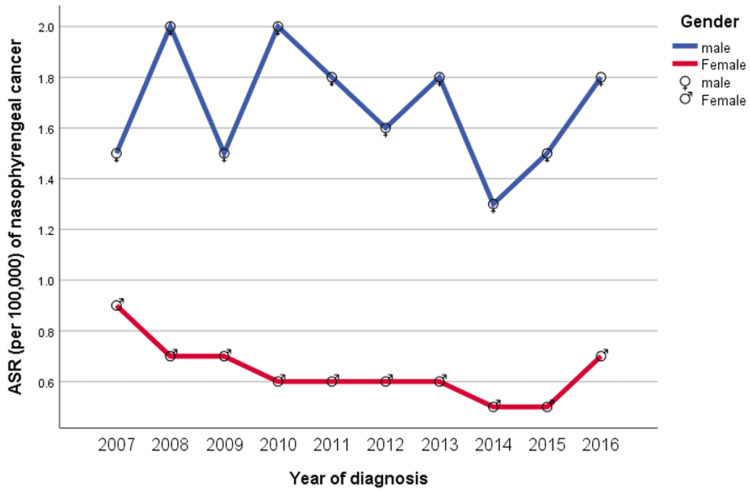
Age-standardized rate (ASR) per 100,000 cases of nasopharyngeal cancer

Between 2007 and 2016, the ASR per 100,000 male cases fluctuated; in 2007, it was 1.5, before falling to 1.3 in 2014, and peaked at 2.0 in 2010 before dropping again to 1.5 in 2015 (Figure 3). The ASR per 100,000 female cases decreased yearly from 0.9 in 2007 to 0.5 in 2015 and then rose to 0.7 in 2016 (Figure 3). In men, the ASR curves, like nasopharyngeal cancer percentages, correlated to the increases and decreases during the study period. In women, the ASR curves gradually decreased over time, remaining constant from 2010 to 2013, and then rose again in 2016 (Figure 3).

Age-specific incidence rate (AIR) of nasopharyngeal cancer increases with age

**Figure 4:**
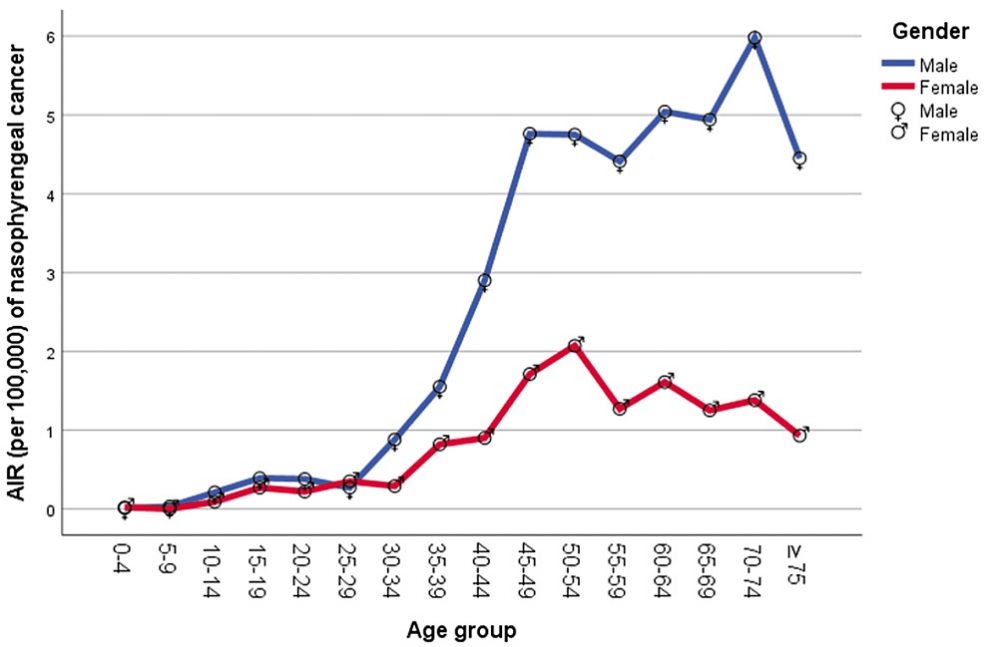
Age-specific incidence rate (AIR) per 100,000 cases of nasopharyngeal cancer

The AIR data from 2007 to 2016 revealed a positive correlation between nasopharyngeal cancer incidence and age, with most cancer cases occurring in older age groups. Figure 4 shows the AIR of nasopharyngeal cancer increasing noticeably from the age of 40 until 74 in men and from 45 to 64 in women, with a peak incidence at menopause age. About 85% of cases were diagnosed at the age of 40 and above.

Age-standardized rate (ASR) of nasopharyngeal cancer varies by region

**Figure 5:**
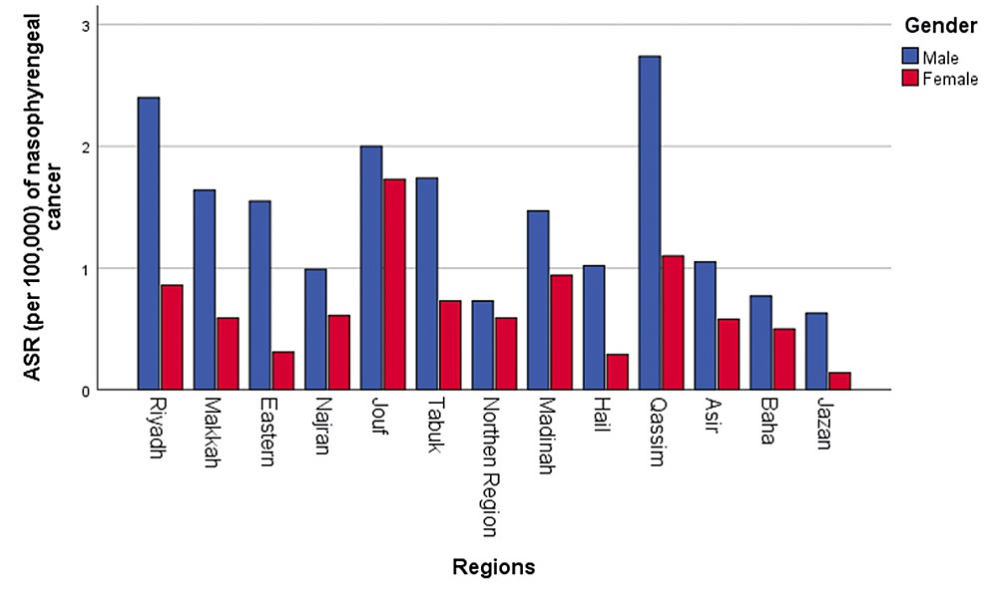
Age-standardized rate (ASR) per 100,000 cases of nasopharyngeal cancer

The ASR data for nasopharyngeal cancer cases demonstrated a wide variation across Saudi regions. The ASR means per 100,000 people from 2007 to 2016 ranged from 0.39 in Jazan to 1.92 in Qassim, with a national average of 1.06 (Figure 5). The Qassim region had the highest male ASR mean at 2.74, followed by the Riyadh region at 2.4 (Figure 5). Conversely, Jazan and northern region provinces reported the lowest ASR averages at 0.63 and 0.73 per 100,000, respectively ​(Figure 5). For women, the Jouf region had the highest ASR mean at 1.73, followed by the Qassim region at 1.1 (Figure 5). Jazan and Hail reported the lowest ASR means at 0.14 and 0.29 per 100,000, respectively (Figure 5).

## Discussion

Nasopharyngeal cancer is an epithelial carcinoma that develops in the mucosa of the nasopharynx [14]. The tumor is frequently detected in the nasopharynx near the pharyngeal recess (fossa of Rosenmüller). Nasopharyngeal cancer and other epithelial head and neck tumors are markedly different despite their shared cell or tissue origins [14-15]. According to the World Health Organization, nasopharyngeal cancer is classified into three pathological subtypes: keratinizing squamous, non-keratinizing squamous, and basaloid squamous [16]. The striking geographical spread of nasopharyngeal cancer incidence has prompted research into its risk factors, and it is suggested that a variety of factors, including Epstein-Barr virus infection, host genetics, smoking, and a history of chronic respiratory tract and environmental factors, all contribute to the development of nasopharyngeal cancer [10,17-19]. The clinical symptoms and indicators of nasopharyngeal cancer coincide with the implicated anatomical regions. Therefore, patients with suspected nasopharyngeal cancer should undergo a thorough head and neck evaluation [14]. Effective population screening can enhance treatment results by detecting people with early-stage disease, which is an appealing option given the scarcity of medical resources in endemic areas such as Southern China [14].

Alrajhi et al. investigate the causes of the delayed diagnosis of nasopharyngeal cancer in Saudi Arabia [11]. They include 307 patients in their study, most of them presenting with advanced stage symptoms (stages III and IV) at the time of evaluation. Overall, 88.6% of them waited for more than three to six months from the onset of symptoms to obtain a diagnosis. Many factors caused the delay, but the most prominent factors were benign conditions, a lack of patient awareness, and a prolonged referral process at that time. The increase in nasopharyngeal cancer cases in recent years should raise the clinical sense of its possibility, especially in the elderly with signs and symptoms in the ear or nose with unclear causes, to prevent delayed diagnosis.

The ASR data revealed that men were diagnosed with nasopharyngeal cancer in Saudi Arabia more often than women, at 2.45 for men and 0.82 for women. This finding concurs with global data showing that males are more suspect to develop nasopharyngeal cancer than women, with an ASR of 2.2 for men and 0.8 for women [13-14]. These rates match the global sex distribution; however, nasopharyngeal cancer (the most prevalent type of head and neck cancer) in Saudi Arabia has a higher overall ASR than the global average, whereas global statistics show that oral cancer is the most common type worldwide [3].

The female ASR decreased gradually from 0.9 in 2007 to 0.5 in 2015 and then rose again to 0.7 in 2016 (Figure 3). In men, the ASR curves, like the nasopharyngeal cancer percentages, correlated to increases and decreases over the study period.

This study, like many others, finds a link between the occurrence of nasopharyngeal cancer and age, with 85% of cases identified at the age of 40 or older. The average age at diagnosis of nasopharyngeal cancer in the United States is 52.7 years, and nearly half of those diagnosed are between the ages of 40 and 59 [3]. Due to prolonged exposure to environmental and behavioral risk factors, aging is associated with greater sensitivity to cancer-causing genetic maturations.

This study found a wide variation in nasopharyngeal cancer incidence in Saudi regions. The results reviewed above found that the Qassim region showed the highest ASR of men with nasopharyngeal cancer, followed by the Riyadh region. In contrast, the Jazan and northern regions presented the lowest ASR. The Jouf region, followed by the Qassim region, had the highest ASR of women with nasopharyngeal cancer. However, Jazan and Hail reported the lowest ASR averages (Figure 5).

Why the nasopharyngeal cancer incidence is higher in the Qassim and Jouf regions but lower in Jazan is unclear. The reason for this could be that Saudis in the Qassim and Jouf regions are more likely to be exposed to unique environmental factors and lifestyle habits. This should be investigated in a future study to identify the risk factors for nasopharyngeal cancer.

This study examined the pattern of nasopharyngeal cancer across Saudi Arabia from 2007 to 2016. The findings of this study may be beneficial for researchers and policymakers in the fields of medicine and healthcare administration in Saudi Arabia as well as for experts in medical and healthcare administration. They could lead to theories about the possible risk and protective factors for nasopharyngeal cancer in the country’s most affected regions. Using this information, epidemiological studies could be conducted to investigate the link between exposure and disease. The data were limited to the Saudi population and did not include other nationalities. Moreover, due to a lack of new reports, the data only ranged from 2007 to 2016.

## Conclusions

Despite a year-to-year fluctuation in the incidence of nasopharyngeal cancer in the Saudi population, there was a significant increase in cases between 2007 and 2016. The positive relation between age and incidence in men and women illustrates that nasopharyngeal cancer is mostly a disease that afflicts the elderly in Saudi Arabia. The considerable variation in incidence rates among Saudi regions raises an important research question about possible causes that seem to be implicated. This study’s findings must be converted into interventions through in-depth assessments of regional differences. This would help prevent nasopharyngeal cancer in Saudi Arabia.
